# Three new species from Guangdong Province of China, and a molecular assessment of *Hygrocybe* subsection *Hygrocybe*

**DOI:** 10.3897/mycokeys.75.59600

**Published:** 2020-12-09

**Authors:** Chao-Qun Wang, Ming Zhang, Tai-Hui Li

**Affiliations:** 1 Guangdong Provincial Key Laboratory of Microbial Culture Collection and Application, State Key Laboratory of Applied Microbiology Southern China, Guangdong Institute of Microbiology, Guangdong Academy of Sciences, Guangzhou 510070, Guangdong, China Guangdong Institute of Microbiology Guangzhou China

**Keywords:** Asia, black discoloration, new taxa, phylogeny, waxcaps

## Abstract

Blackening waxcaps (Hygrocybe subsect. Hygrocybe) are a group of colorful and attractive mushrooms. However, the species diversity of subsect. Hygrocybe in China is still poorly known due to the limited sampling. In this study, three new species of this group from Guangdong Province, China are described and illustrated based on their morphological characteristics and molecular analyses of the internal transcribed spacer and large subunit ribosomal DNA regions. *Hygrocybe debilipes* from grasslands of South China Sea islands is mainly characterized by its orange red to vivid red pileus, fragile stipe, and ellipsoid to oblong basidiospores; *H. griseonigricans* from woodlands is characterized by its whitish to dull yellow pileus, quick black discoloration and the globose, subglobose to broadly ovoid basidiospores; *H. rubroconica* from woodlands is characterized by the hemispheric to plano-convex pileus when mature, semitranslucent fibrose stipe, and globose to ellipsoid basidiospores.

## Introduction

The type species of genus *Hygrocybe* (Fr.) P. Kumm., *H. conica* (Schaeff.) P. Kumm., belongs to subgen. Hygrocybe (Fr.) P. Kumm., sect. Hygrocybe (Fr.) P. Kumm., subsect. Hygrocybe (Fr.) P. Kumm. (Lodge et al. 2014). Taxa of subsect. Hygrocybe (*H. conica*-complex) are widely distributed, common and attractive. Morphologically, the group is mainly characterized by the blackening basidiomata, conical and usually fibrillose pileus, free to slightly attached lamellae, parallel and long hymenophoral trama hyphae usually over 200 μm long, and length ratio of basidium to basidiospore usually less than 5 (Lodge et al. 2014). Phylogenetically, members of subsect. Hygrocybe are clustered as a monophyletic group using the internal transcribed spacer (ITS) and/or the nuclear large subunit ribosomal DNA (LSU) as gene markers (Babos et al. 2011; Lodge et al. 2014; Vizzini et al. 2015; Deepnalatha and Manimohan 2018; Wang et al. 2019). Ecologically, they are positive environmental indicators in ecosystems since they typically grow in less polluted grasslands or woodlands (Griffith et al. 2002; Wood and Dunkelman 2017).

To date, there are 25 taxa in the world that fit the morphological characteristics of subsect. Hygrocybe. Ten taxa under the group have been originally described from Europe: *H. cinereifolia* Courtec. & Priou and *H. nigrescens* (Quél.) Kühner from France, *H. conica* from Germany, H. conica var. aurantiolutea T. Borgen & Arnolds from Greenland, H. conica var. conicopalustris (Bon) Arnolds and H. conica var. minor Monthoux & Röllin from Switzerland, *H. conicoides* (P.D. Orton) P.D. Orton & Watling and *H. olivaceonigra* (P.D. Orton) M.M. Moser from the UK (England), *H. pseudoconica* J.E. Lange from Denmark, and *H. veselskyi* Singer & Kuthan from Czech Republic (Kummer 1871; Lange 1923; Kühner 1926; Moser 1967; Orton and Watling 1969; Singer and Kuthan 1976; Arnolds 1986; Courtecuisse 1992; Monthoux and Röllin 1993; Borgen and Arnolds 2004). Five taxa were originally described from Africa; they are *H. astatogala* (R. Heim) Heinem. from Madagascar, H. astatogala var. laeticolor Heinem., H. conica var. pallidipes Heinem. and *H. cortinata* Heinem. from Zaire, and *H. chloroides* (Malançon) Kovalenko from Marocco (Heinemann 1963; Kovalenko 1989). Five taxa have been originally described from North America, including *H. albifolia* (Hesler & A.H. Sm.) R. Valenz., Guzmán & J. Castillo, *H. foliirubens* Murrill, *H. singeri* (A.H. Sm. & Hesler) Singer and *H. cuspidata* (Peck) Murrill from the USA, and H. conica var. atrosanguinea (Grund & K.A. Harrison) Malloch from Canada (Nova Scotia) (Murrill 1941; Singer 1958; Valenzuela et al. 1981; Malloch 2010). Two taxa have been originally described from Asia, H. conica var. peradenyca (Sacc.) Pegler from Sri Lanka and *H. erinacea* (Pat.) Singer from Vietnam (Singer 1958; Pegler 1986). Two taxa were described from the Caribbean Region, *H. atrosquamosa* Pegler from Martinique and H. conica var. brevispora (Dennis) S.A. Cantrell & Lodge from Venezuela (Pegler 1983; Cantrell and Lodge 2000). In addition, H. conica var. tierneyi A.M. Young was originally described from Australia (Young and Wood 1997).

The existing knowledge about blackening *Hygrocybe* in China is still rather limited. There have been no phylogenetic studies based on Chinese specimens until now. No new species of subsect. Hygrocybe has been described from China, while the Chinese samples of this group are commonly treated as European species, such as *H. conica* and *H. nigrescens* (Chen and Li 2013).

Guangdong Province, which is located in South China, belongs to the East Asian monsoon region. The climate can be divided into the middle subtropical, the southern subtropical, and the tropical climate zones, from north to south. The annual average temperature is 19–24 °C and the average annual precipitation is 1300–2500 mm. During the authors’ field trips over the past ten years, numerous samples of blackening waxcaps with diverse morphological characteristics have been found. Obviously, the species diversity of subsect. Hygrocybe in Guangdong Province has been underestimated in the past.

In this study, a new worldwide phylogenetic framework of subsect. Hygrocybe is reconstructed using the ITS and LSU regions. Three new species from Guangdong Province are described based on the morphological characteristics, molecular phylogenetic evidence, and ecological data.

## Materials and methods

### Sampling and morphological studies

For each collection, fresh specimens were observed, photographed, and collected in situ; the date, location, collector, elevation, habitat and macroscopic characteristics were documented, and then, the specimens were dried below 50 °C overnight in an electric dryer. Macroscopic descriptions were based on the field records and colored photos. Color descriptions and codes refer to Kornerup and Wanscher (1978). Sizes and shapes of basidiospores, basidia, pileipellis, stipitipellis, and hymenophoral trama were observed using handmade tissues stained with 5% potassium hydroxide solution and/or 1% Congo red solution under an AX10 light microscope and photographed using ZEN 2 lite software (Zeiss, Oberkochen, Germany). The detailed measurement method has been described by Wang et al. (2020). Specimens are deposited in the Fungarium of Guangdong Institute of Microbiology, Guangzhou, China (GDGM).

### DNA extraction, PCR amplification and sequencing

Genomic DNAs were extracted from a small amount of dry lamellar tissue using the HiPure Fungus DNA Mini kit (Magen Biotech, Guangzhou, China). ITS and LSU gene regions were amplified using Polymerase chain reactions (PCR) with primers ITS1/ITS1F/ITS5 and ITS4 (White et al. 1990; Gardes and Bruns 1993), LR0R and LR5/LR7 (https://sites.duke.edu/vilgalyslab/rdna_primers_for_fungi/), respectively. Bidirectional PCR product sequencing was carried out using the same primers that were used in the PCR reactions. The forward and reverse raw sequences were assembled and trimmed using SeqMan version 7.1.0 (DNAStar, Inc.). The consensus sequences were blasted in the National Center for Biotechnology Information (NCBI) to rule out the possibility of contamination, and then the correct consensus sequences were deposited in the International Nucleotide Database [accession numbers: MW001782–MW001792 (ITS), MW007875–MW007884 (LSU)].

### Phylogenetic analyses

To elucidate the phylogenetic position of the fungal samples with new sequences within the genus *Hygrocybe*, the newly obtained and all the available *Hygrocybe*LSU sequences in NCBI were included. In addition, the sequences of *Hygroaster albellus* Singer (EF551314), *H. nodulisporus* (Dennis) Singer (EF561625), and “*H. andersonii* Cibula & N.S. Weber” (KF291171) were selected as the outgroup, according to Vizzini et al. (2015). To clarify the phylogenetic position of the new sequences within subsect. Hygrocybe, the newly gained and all usable ITS sequences of sect. Hygrocybe were included, and members of subsect. Macrosporae were treated as the outgroup, according to Lodge et al. (2014) and Wang et al. (2019). To make LSU and ITS matrixes, the new sequences and the downloaded reference sequences were first combined, aligned using MAFFT online service (Katoh et al. 2017) by applying the auto strategy, and then viewed and trimmed (the front and back parts) using MEGA-X software (Kumar et al. 2018). The data alignments have been submitted to TreeBASE, submission ID 27252 (LSU) and 27253 (ITS). The substitution models were selected using the Bayesian information criterion with ModelFinder (Kalyaanamoorthy et al. 2017) in PhyloSuite (Zhang et al. 2020). Phylogenetic analysis for each dataset was performed using the maximum likelihood (ML) in IQ-TREE (Nguyen et al. 2015); and the node bootstrap support values (BS) were estimated using an ultrafast bootstrap with 5000 replicates (Minh et al. 2013). Phylograms were viewed and annotated with iTOL version 5.5 (Letunic and Bork 2016).

## Results

### Phylogenetic analyses

The LSU alignment contains 138 sequences with 1067 columns, of which 611 are constant sites and 333 are parsimony informative sites. The model of substitution is TIM3e+R4 according to Bayesian Information Criterion using ModelFinder. The ITS alignment has 103 sequences with 772 columns, of which 380 are constant sites and 294 are parsimony informative sites. K3Pu+F+G4 is selected as the best-fit model for the ITS dataset.

**Table 1.** Taxa, vouchers, geographic origins and sequence accession numbers of newly generated sequences.

Taxon	Voucher	Geographic origin	ITS	LSU
*H. debilipes*	GDGM59314	China: Guangdong	MW001783	MW007877
	GDGM57013	China: Guangdong	MW001782	MW007875
	GDGM59131	China: Guangdong	MW001785	–
	GDGM59133	China: Guangdong	MW001784	MW007876
*H. griseonigricans*	GDGM73527	China: Guangdong	MW001790	MW007883
	GDGM81385	China: Guangdong	MW001791	MW007882
	GDGM81394	China: Guangdong	MW001792	MW007884
*H. rubroconica*	GDGM45213	China: Guangdong	MW001786	MW007881
	GDGM44706	China: Hunan	MW001788	MW007878
	GDGM45209	China: Guangdong	MW001787	MW007879
	GDGM45214	China: Guangdong	MW001789	MW007880

**Figure 1.**
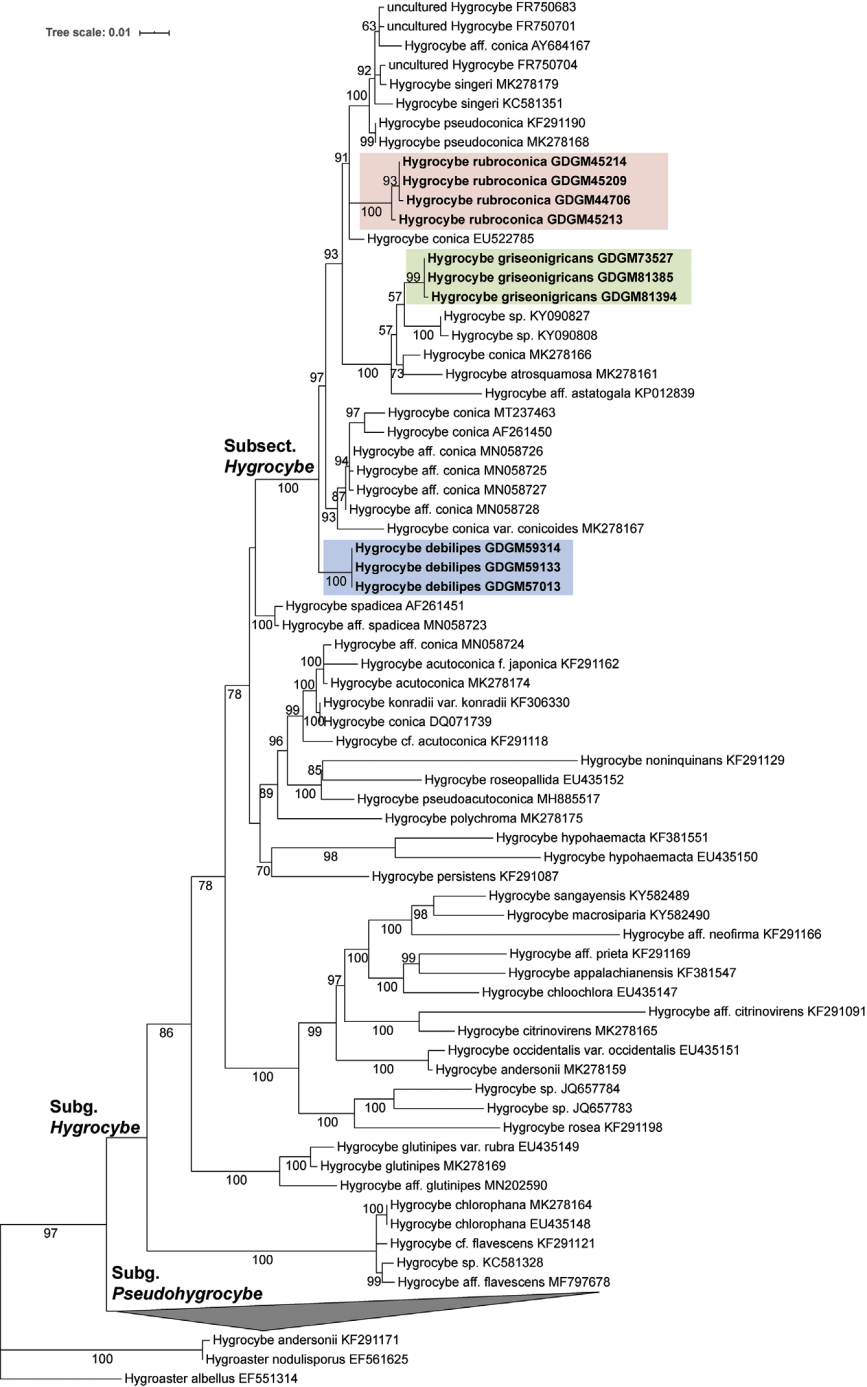
Maximum Likelihood phylogram of genus *Hygrocybe* based on LSU matrix, rooted with *Hygroaster albellus* (EF551314), *H. nodulisporus* (EF561625) and *H. andersonii* (KF291171). The newly generated sequences in this study are showed in bold. GenBank accession numbers of downloaded sequences or voucher numbers of new sequences are given after the species names. Bootstrap values more than 50% are shown around the branches.

In the LSU phylogram (Fig. 1), genus *Hygrocybe* [except for “*H. andersonii*” (KF291171)] forms a strongly supported monophyletic clade with 97% BS; *Hygroaster nodulisporus* (EF561625) and “*H. andersonii*” (KF291171) are clustered (100% BS), in accordance with the LSU analysis of Vizzini et al. (2015). The division of *Hygrocybe* into two subgenera is strongly supported (97% BS). Ten newly generated sequences are present as members of subsect. Hygrocybe: three sequences of *H. debilipes* are located at the most distal part of subsect. Hygrocybe with 100% BS; three sequences of *H. griseonigricans* appear as a sister clade to an undescribed species (KY090808 and KY090827) with limited support (57% BS) within a larger clade containing H. aff. astatogala (KP012839) and *H. atrosquamosa* (MK278161); four sequences of *H. rubroconica* form an independent clade with high support (100% BS).

**Figure 2.**
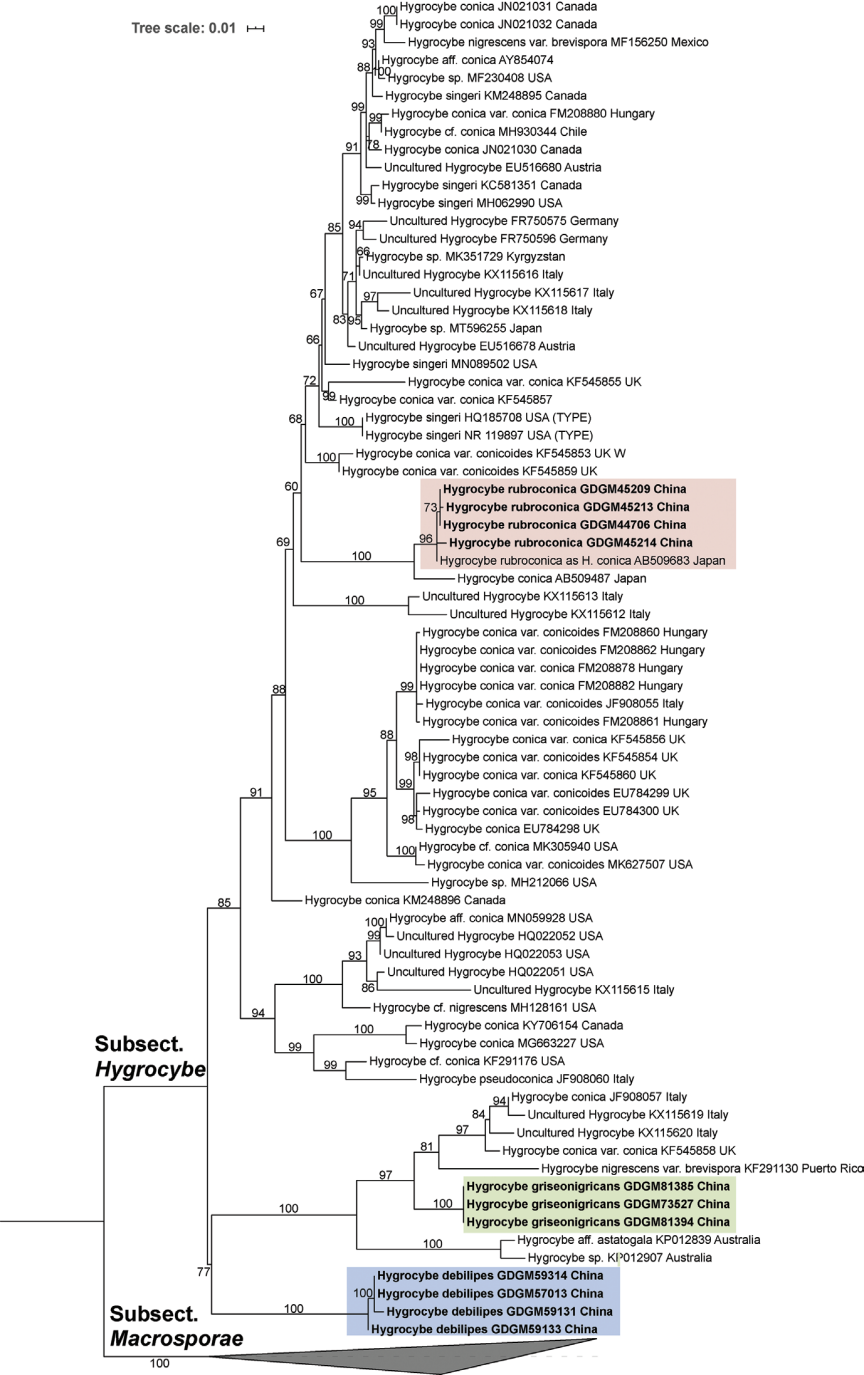
Maximum-likelihood tree of sect. Hygrocybe based on ITS matrix, rooted with members of subsect. Macrosporae. The newly generated sequences in this study are indicated in bold face. GenBank accession number of downloaded sequence or voucher number of newly generated sequence and place of origin are given after the species name of each sequence. Bootstrap values more than 50% are shown around the branches.

In the ITS phylogram (Fig. 2), members of subsect. Hygrocybe and subsect. Macrosporae are treated as the ingroups and outgroups, respectively. Although all new species are present in subsect. Hygrocybe, the ITS analysis produces partially different typologies from the LSU analysis regarding the relationships of taxa under subsect. Hygrocybe. In the ITS analysis, four new sequences of *H. debilipes*, three new sequences of *H. griseonigricans*, and five sequences [four newly from China and one from Japan labeled as “*H. conica*” (AB509683)] of *H. rubroconica* form three independent clades, respectively.

### Taxonomy

#### Hygrocybe debilipes

Taxon classificationFungiAgaricalesHygrophoraceae

C.Q. Wang & T.H. Li sp. nov.

96F62879-6B0F-5C75-9C65-3097624A456A

836234

Figures 3A, B , 4

##### Typification.

China. Guangdong Province, Taishan City, Chuandao Town, Xiachuan Island, on a grassland, elev. ca. 50 m, 21°37'36"N, 112°34'30"E, 17 July 2017, H. Huang, Q.J. Huang & X.R. Zhong (GDGM59314, holotype!).

##### Sequences ex holotype.

MW001783 (ITS), MW007877 (LSU).

##### Etymology.

“*debili*-”: not strong, “-*pes*”: stipe. The species epithet “*debilipes*” (Lat.) refers to the fragile stipe of the new species.

##### Diagnosis.

*Hygrocybe debilipes* differs from *H. singeri* in having a smaller pileus, orange red to vivid red pileus before discoloration, pale yellow to light orange lamellae, a fragile and less sticky stipe, ellipsoid to oblong basidiospores, and the distribution in South China Sea islands.

##### Description.

Pileus 5–12 mm diam., conical with an acute umbo when young, convex to hemispherical in age, inrolled at margin, orange red (8A7–8), red to vivid red (9A7–8, 9B7–8), blackening with injury or aging, sticky when moist, hygrophanous. Lamellae free, pale yellow (4A3) to light orange (5A4) when mature, blackening with injury or aging, up to 4 mm broad, distant, waxy, with 1–3 unequal lamellulae between two entire lamellae, with lighter color at lamellar edge. Stipe 22–45 × 2–5 mm, central, cylindrical, equal or slightly tapered at apex, fistulous, semitranslucent, usually too fragile to obtain a complete stipe base, pale yellow (4A3) to light orange (5A4) with white base, blackening with injury or aging, glabrous to fibrillose, moist.

Basidiospores (7.5)8–11.5(12) × (4.5)5–7(7.5) μm [mean length = 9.4 μm, mean width = 6 μm], Q = 1.3–1.8, Q_m_ = 1.6, ellipsoid to oblong, smooth, thin-walled, hyaline. Basidia 32–53 × 8–13 μm, 4-spored, clavate, with sterigmata up to 7 μm long. Pileipellis a cutis of cylindrical hyphae 2–11 μm diam., thin-walled, hyaline. Stipitipellis a cutis or ixocutis of repent, thin-walled, clamped hyphae 2.5–16 μm diam. Hymenophoral trama regular to subregular, consisting of parallel hyphal cells 4–17 μm diam., thin-walled, hyaline.

##### Habit, habitat and distribution.

Scattered on grasslands in summer. Known from South China Sea islands.

##### Additional specimens examined.

China. Guangdong Province, Taishan City, Chuandao Town, Xiachuan Island, elev. ca. 50 m, 17 July 2017, Q.J. Huang, H. Huang & X.R. Zhong (GDGM59131, GDGM59133); Zhuhai City, Dawanshan Island, 28 July 2013, T.H. Li, H. Huang & Y.W. Xia (GDGM57013).

##### Remarks.

*Hygrocybe debilipes* is morphologically and genetically a distinct species. *Hygrocybe debilipes* is characterized by its tiny basidioma, orange red to vivid red pileus, pale yellow to light orange lamellae when mature, fragile and semitranslucent stipe, and ellipsoid to oblong basidiospores measuring (7.5)8–11.5(12) × (4.5)5–7(7.5) μm. *Hygrocybe debilipes* forms a strongly supported independent clade in both ITS and LSU phylogeny trees (Figs 1–2).

*Hygrocybe cinereifolia*, originally described from France, is morphologically similar to *H. debilipes* in its general appearance. However, *H. cinereifolia* has larger basidiomata and grayish to gray lamellae (Courtecuisse 1992, http://www.pharmanatur.com/Mycologie/Hygrocybe%20cinereifolia.htm). *Hygrocybe singeri*, originally described from northwestern USA, is also morphologically similar to *H. debilipes*. However, *H. singeri* is larger (10–50 mm diam.), and possesses a yellow to orange pileus and greenish yellow lamellae (Hesler and Smith 1963).

#### Hygrocybe griseonigricans

Taxon classificationFungiAgaricalesHygrophoraceae

C.Q. Wang & T.H. Li sp. nov.

5B886BDB-2EE1-551C-B20F-9ED7708E346F

836235

Figures 3C, D , 5

##### Typification.

China. Guangdong Province, Nanxiong City, Qingzhang Mountain, elev. ca. 340 m, 15 May 2018, M. Zhang & X.N. Chen (GDGM73527, holotype!).

##### Sequences ex holotype.

MW001790 (ITS), MW007883 (LSU).

##### Etymology.

“*griseo*-”: gray, “-*nigricans*”: black. The species epithet “*griseonigricans*” refers to its gray pileus with obvious blackening reaction.

##### Diagnosis.

*Hygrocybe griseonigricans* differs from *H. astatogala* by having a duller pilus color, variable (subglobose to elongate) basidiopore shapes, 1-, 2- and 4-spored basidia and differences in molecular sequences.

##### Description.

Pileus 25–70 mm diam., broad conical to umbonate disc when young, expanded to umbonate, convex, even to almost plane when mature, white, pale yellow (3A3) to dull yellow (3B3), densely covered with radially arranged black hairy fibrils with appressed or uplifted ends on surface, nigrescent when bruised or mature; margin incurved when young, expanded to straight when mature. Lamellae free, white at first, turning black when bruised or mature, up to 7 mm wide, waxy, fragile, with 1–3 lamelluate between two entire lamellae, edge usually eroded. Stipe 50–150 × 5–12 mm, central, cylindrical, sometimes slightly curve, usually wider at base, hollow, white to yellow at the upper part, usually white at the base, changing to black with age or when bruised, covered with clustered black longitudinal fibrils.

Basidiospores 9–10.5 × (6)6.5–9.5(10) μm [mean length = 9.7 μm, mean width = 8 μm], Q = (0.95)1.1–1.6, Q_m_ = 1.23, globose, subglobose to broadly ovoid, smooth, thin-walled. Basidia 32–44.5 × 8–11.5 μm, 1-, 2- or 4-spored, clavate, with sterigmata up to 10 μm long, sterigmata of 2-spored basidia usually longer than those of 4-spored basidia. Pileipellis a cutis or trichoderm, hyphae 2–15 μm in diam. Hymenophoral trama regular, hyphae 2.5–21 μm broad, translucent, mainly thin-walled rarely thick-walled.

##### Habit, habitat and distribution.

Solitary to scattered on soil; basidiomata occurring from April to September. So far known from Southern China.

##### Additional specimens examined.

China. Guangdong Province, Nanxiong City, Qingzhang Mountain, elev. ca. 340 m, 15 May 2018, M. Zhang & X.N. Chen (GDGM73528); Renhua County, Danxia Mountain, elev. ca. 100 m, 12 June 2020, M. Zhang & L.Q. Wu (GDGM81385).

##### Remarks.

The distinctive morphological features of *Hygrocybe griseonigricans* are the following: white to dull yellow-colored pileus with black discoloration, white lamellae, white stipe base, and variable basidiospore shapes, from globose, subglobose to broadly ovoid. The ITS and LSU phylogenetic analyses support *H. griseonigricans* as a distinct species within a well-supported clade that includes *H. astatogala* (Figs 1–2).

*Hygrocybe conica*, originally described from Germany, is similar to *H. griseonigricans*. However, *H. conica* has a larger (up to 100 mm diameter) and somewhat fibrillose pileus, and usually grayish lamellae (Boertmann 2010). *Hygrocybe astatogala*, originally described from Madagascar, resembles *H. griseonigricans* in the black fibrils on the pileus. However, *H. astatogala*, has distinct and persistent conical and yellow to orange pileus, yellow to orange lamellae, and subglobose to ovoid basidiospores (Horak 1990; Leelavathy et al. 2006).

**Figure 3.**
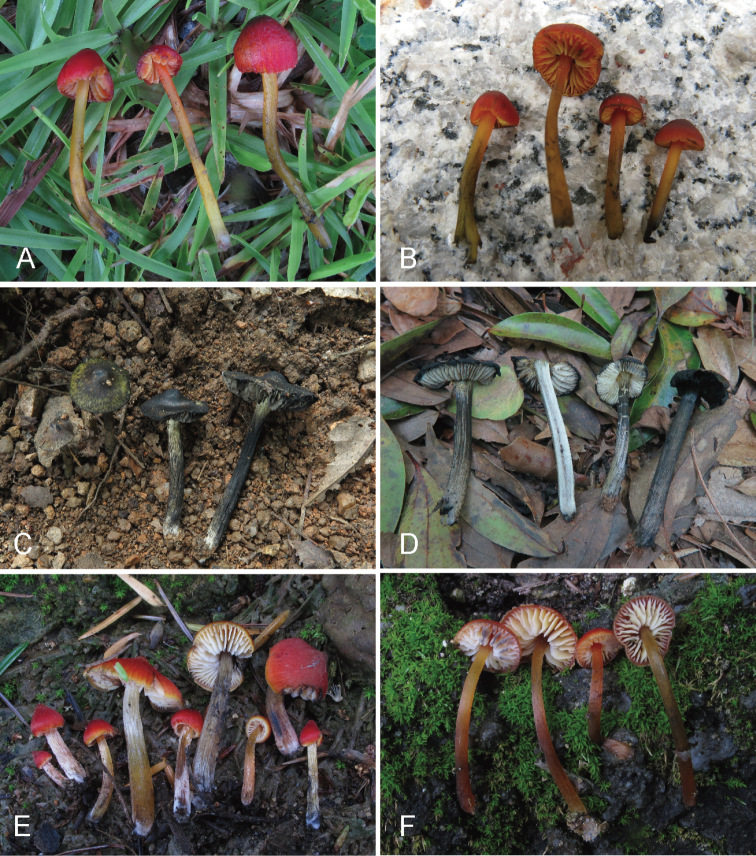
Basidiomes of *Hygrocybe* species **A***Hygrocybe debilipes* (GDGM59314) **B***Hygrocybe debilipes* (GDGM57013) **C***Hygrocybe griseonigricans* (GDGM73527) **D***Hygrocybe griseonigricans* (GDGM81394) **E***Hygrocybe rubroconica* (GDGM45213) **F***Hygrocybe rubroconica* (GDGM45214).

**Figure 4.**
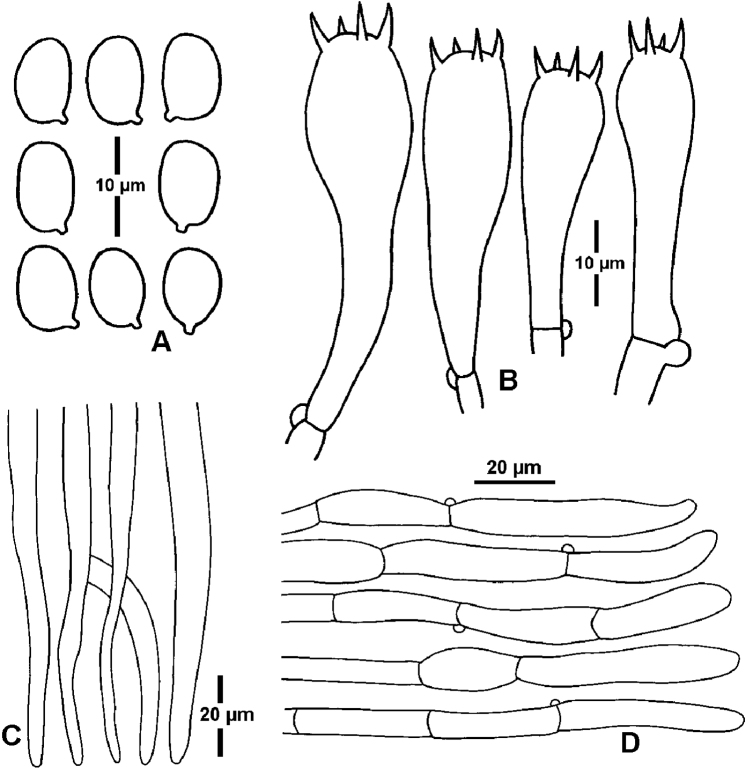
Microscopic features *Hygrocybe debilipes* (GDGM59314) **A** basidiospores **B** basidia **C** terminal cells of hymenophoral trama **D** terminal cells of pileipellis.

**Figure 5.**
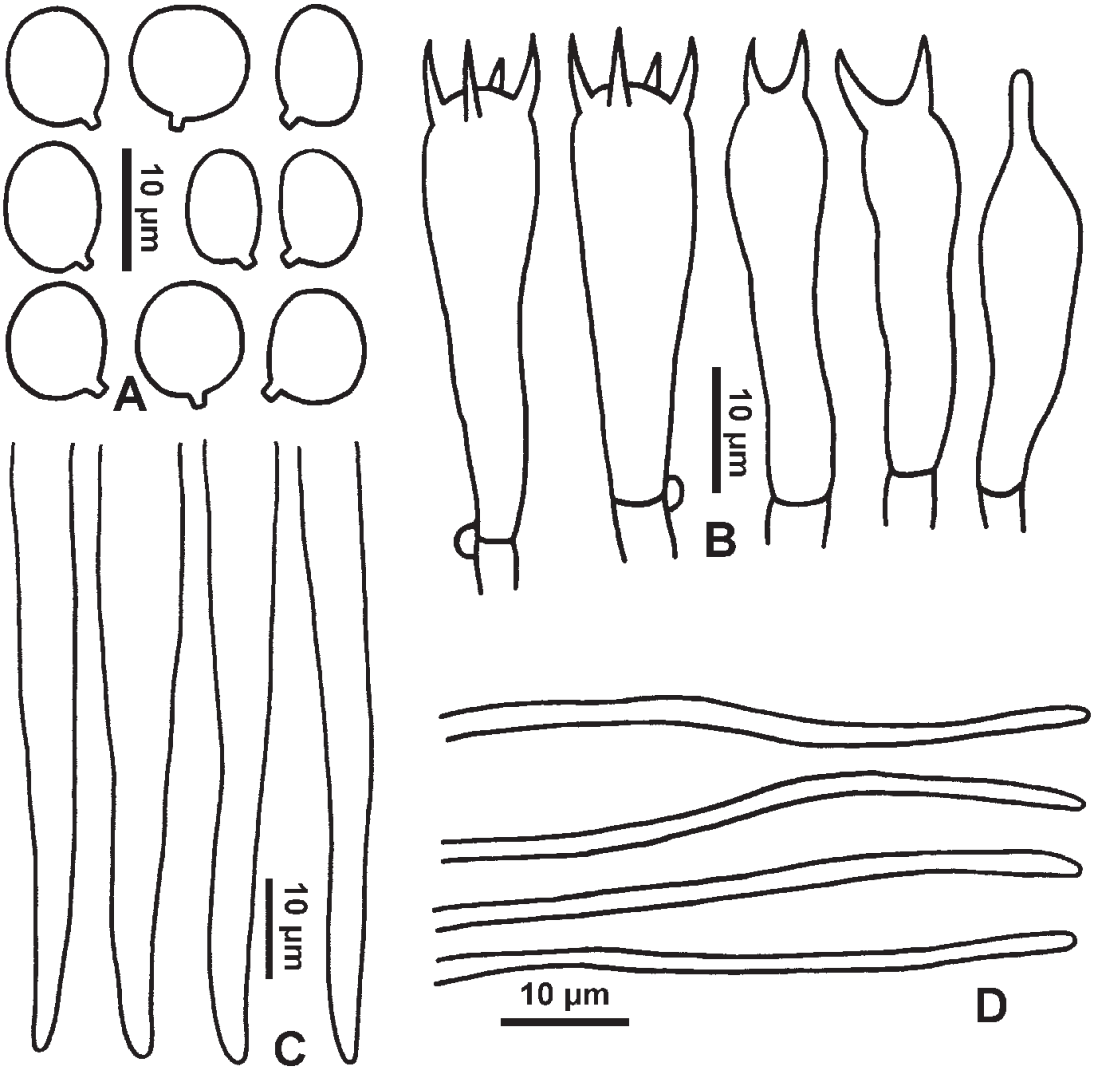
Microscopic features *Hygrocybe griseonigricans* (GDGM73527) **A** basidiospores **B** basidia **C** terminal cells of hymenophoral trama **D** terminal cells of pileipellis.

#### Hygrocybe rubroconica

Taxon classificationFungiAgaricalesHygrophoraceae

C.Q. Wang & T.H. Li sp. nov.

A577A0A3-68AB-502B-AC4B-8513B8DF2733

837541

Figures 3E, F , 6

##### Typification.

China. Guangdong Province, Shaoguan City, Ruyang County, Nanling Natural Reserve, elev. ca 1180 m, 15 May 2015, Ming Zhang, Hao Huang and Jiang Xu (GDGM45213, Holotype!).

##### Sequences ex holotype.

MW001786 (ITS), MW007881 (LSU).

##### Etymology.

“*rubro*-”: red, “-*conica*”: conical. The species epithet “*rubroconica*” refers to its red pileus surface.

##### Diagnosis.

*Hygrocybe rubroconica* differs from *H. conica* by having redder pileus, white to light reddish white and ventricose to broadly ventricose lamellae, simitranslucent stipe covered with white fibrils at first, and mainly 2-spored basidia.

##### Description.

Pileus 5–25 mm broad, conical, conical when young, broadly conical, hemisphere to plano-convex when mature, sometimes with an umbo in center, red to vivid red (10A7–8, 10B7–8), usually darker at disc, translucently striate from the margin to about a half of radius, blackening when bruised or mature; margin incurved, white to light yellow to reddish yellow (4A5–6). Lamellae free, ventricose to broadly ventricose, white to yellowish white, blackening when bruised or mature, up to 4 mm broad, distinct, waxy, fragile, with 1–3 lamellula between two entire lamellae. Stipe 20–40 mm long, 1–4 mm thick, central, cylindrical, slightly enlarged towards the base, hollow, simitranslucent, pale yellow (4A3) to brownish orange (5C3–5), nigrescent when bruised or old, usually covered with white longitudinal fine fibrils when young.

**Figure 6.**
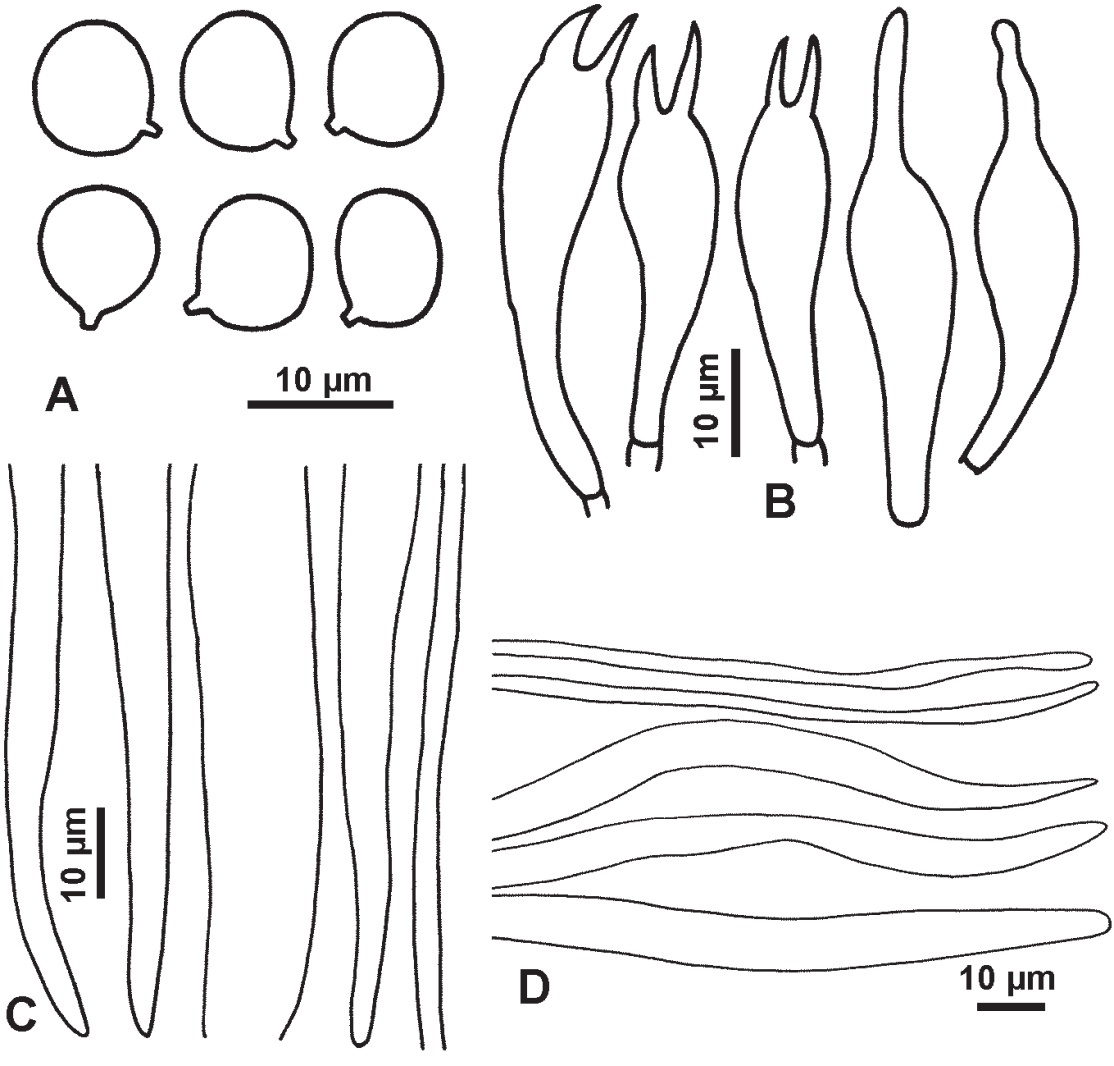
Microscopic features *Hygrocybe rubroconica* (GDGM45213) **A** basidiospores **B** basidia **C** Hymenophoral trama cells **D** terminal cells of pileipellis.

Basidiospores (7.5)8–10.5(11) × (6.5)7–8.5 μm [mean length = 9 μm, mean width = 7.6 μm], Q = 1–1.47, Q_m_ = 1.2, globose, subglobose, broadly ellipsoid to ellipsoid, smooth, thin-walled. Basidia 25.5–45 × 8.5–12 μm, mainly 2-spored, rarely 4-spored, clavate, containing some black endochrome in KOH, with sterigmata up to 10.5 μm long. Pseudocystidia present. Pileipellis as a cutis, comprised of parallel hyphae 4–10 μm wide, occasionally with bend hyphae, with some black inclusions. Stipipellis made up of parallel hyphae, occasionally with some thick-walled haphae. Hymenophoral trama regular, of hyaline and thin-walled hyphae 8.5–17 μm wide, sometimes containing blackish brown materials.

##### Habit, habitat and distribution.

Scattered to clustered on moist soil in forests; occurring from April to September. So far only known from Southern China.

##### Additional specimens examined.

China. Guangdong Province, Ruyang County, Nanling Natural Reserve, elev. ca. 1400 m, 24°89'N, 113°01'E, 16 June 2014, M. Zhang & T. Li (GDGM45209); same location, 15 May 2015, M. Zhang, H. Huang & J. Xu (GDGM45214).

##### Remarks.

*Hygrocye rubroconica* is characterized by the red pileus, ventricose to broadly ventricose lamellae, nearly translucent stipe covered with white fibrils at first, globose to ellipsoid basidiospores, mainly 2-spored basidia, and the presence of pseudocystidia.

*Hygrocybe veselskyi* Singer & Kuthan, originally described from Czechoslovakia, resembles *H. rubroconica* in its general appearance due to the red pileus and black staining reaction when touched or mature; however, *H. veselskyi* differs from *H. rubroconica* since it has yellow lamellae and bigger basidiospores measuring 10–12.5 × 5.3–6 μm (Candusso 1997).

## Discussion

In this study, three new species of Hygrocybe subsect. Hygrocybe from Guangdong Province, China are described and compared with similar species based on morphological and molecular data. More comprehensive phylogeny of genus *Hygrocybe* (focusing on subg. Hygrocybe) based on the LSU sequences and of sect. Hygrocybe based on ITS sequences are provided, including almost all the representatives in GenBank database and newly generated sequences. This study not only provides new species and genetic information of subsect. Hygrocybe from East Asia, but also provide some ideas on the species delimitation of subsect. Hygrocybe based on morphological and phylogenetic evidences.

Morphologically, the sizes of both fresh and dried basidiomata of *H. griseonigricans*, *H. rubroconica* and *H. debilipes* decrease in turns. The stipe of *H. debilipes* is especially more fragile than that of *H. griseonigricans* or *H. rubroconica*. Although the pileus color is variable within a species, the pileus color range, the pileus blackening rate and degree, and the lamellar color can be used as distinguishing features for distinguishing the three new species in southern China. For example, *H. debilipes* and *H. rubroconica* have a red to orange pileus, while *H. griseonigricans* has a white to yellow pileus. The pileus of *H. griseonigricans* mostly becomes black when mature, while the pileus of *H. debilipes* and *H. rubroconica* seldom becomes black when mature. The lamellar color of *H. debilipes* is orange, while *H. griseonigricans* and *H. rubroconica* have almost white lamellae. In contrast with the macro-morphology, limited micro-morphological features can be used to discriminate the species within subsect. Hygrocybe. The intraspecific variations in the basidiospore shape and in the number of basidum sterigmata are small in *H. debilipes*, but large in *H. griseonigricans* and *H. rubroconica*.

Molecular analyses seems to provide more stable information for species identification of subsect. Hygrocybe. Taking *H. singeri* as an example (Fig. 2), due to the release of the type specimen’s ITS sequences in GenBank (NR_119897 and HQ185708), it is clear that the sequences retrieved from several samples that are labeled as “*H. singeri*” (KM248895, KC581351, MH062990 and MN089502) should be a different species. However, the shortage of released correctly identified sequences is a basic issue that needs to be urgently addressed. Within subsect. Hygrocybe, it is highly urgent to obtain the sequences from a well-identified *H. conica* specimen from the type location at Bavaria, Germany, since *H. conica* has undergone various interpretations in different continents (Figs 1–2).

To reach a point in which all validly published species names of blackening waxcaps are represented by pertinent DNA sequences, it is necessary to obtain sequences from well-identified specimens of existing species. These tasks may not be completed in a short time since specimens from the type location need to be found by the researchers, amateurs, or society citizens, and then carefully identified by fungal taxonomists with relevant research experience. In addition, new species should be published based on both morphological and molecular data.

## Supplementary Material

XML Treatment for Hygrocybe debilipes

XML Treatment for Hygrocybe griseonigricans

XML Treatment for Hygrocybe rubroconica
